# Graphene Oxide Nanoparticles Induce Apoptosis in wild-type and CRISPR/Cas9-IGF/IGFBP3 knocked-out Osteosarcoma Cells

**DOI:** 10.7150/jca.46464

**Published:** 2020-06-21

**Authors:** Mervin Burnett, Yasser Abuetabh, Ania Wronski, Fan Shen, Sujata Persad, Roger Leng, David Eisenstat, Consolato Sergi

**Affiliations:** 1 Department of Laboratory Medicine and Pathology, Stollery Children's Hospital, University of Alberta, Edmonton, Alberta, Canada.; 2 Department of Laboratory Medicine and Pathology, Heritage Medical Research Centre, University of Alberta, Edmonton, Alberta, Canada.; 3 Synthego Corporation, Menlo Park, CA 94025, USA.; 4 Department of Paediatrics, University of Alberta, Edmonton, Alberta, Canada.; 5 Department of Medical Genetics, University of Alberta, Edmonton, Alberta, Canada.; 6 Department of Oncology, University of Alberta, Edmonton, Alberta, Canada.; 7 National "111" Center for Cellular Regulation and Molecular Pharmaceutics, Key Laboratory of Fermentation Engineering (Ministry of Education), Hubei University of Technology, Wuhan 430068, P.R. China.; 8 Department of Orthopedics, Tianyou Hospital, Wuhan University of Science and Technology, Wuhan, Hubei, P.R. China.

**Keywords:** Osteosarcoma, cell line, apoptosis, ROS, CRISPR-Cas9, IGF1, IGFBP3, Graphene oxide

## Abstract

Osteosarcoma affects both adolescents and adults, and some improvement in the survival rate for affected patients has been reached in the last decade. Still, non-specificity and systemic toxicity may limit traditional therapeutic approaches to some extent. The insulin growth factor 1 (*IGF1*) and its binding protein (*IGFBP3*) have been implicated in the tumorigenesis. Nanoparticles, such as graphene oxide (GO), can provide an effective treatment for cancer as they can specifically target cancer cells while reducing undesired side effects. This study aimed to evaluate the toxicity of GO on osteosarcoma *in vitro* using tumor cell lines with and without knocking out the *IGF* and *IGFBP3* genes. Human osteosarcoma cell lines, U2OS and SAOS2, and the normal osteoblast cell line hFOB1.19 were used. The *IGF1* and *IGFBP3* genes were eliminated using CRISPR/Cas9. Tumor cells were cultured and treated with GO. Apoptosis and reactive oxygen species (ROS) were analyzed by Annexin V-FITC and ROS assays. The nuclear factor erythroid 2-related factor 2 (NRF2), which is a crucial regulator of cellular resistance to oxidants, was investigated by Western blotting. We found a significantly higher rate of apoptosis in the OS than hFOB1.19, especially in U2OS cells in which *IGF1* and *IGFBP3* were knocked out. ROS increase due to GO exposure was remarkably time and concentration-dependent. Based on the rate of apoptosis, ROS, Nrf-2 decrease, and cytomorphological changes, GO has a significant cytotoxic effect against OS. Targeting the *IGF1* and *IGFBP3* signaling pathway may strengthen GO-related cytotoxicity with the potential to increase the survival of patients affected by this tumor.

## Introduction

An improvement in the survival rate for cancer-affected persons has characterized the scientific scene of the last two decades. However, there is an urgent need to develop newer treatment options to maximize the outcome of cancer therapy and to minimize long-term toxicities in cancer survivors 1. Osteosarcoma (OS) has a bimodal distribution pattern occurring in both adolescents and the elderly. It comprises about 20% of primary bone sarcomas 2, 3. The pediatric age group is most affected as the peak incidence of the OS tumor coincides with the period of rapid skeletal growth. OS is slightly more prevalent in males and may arise in any bone with the appendicular skeleton mainly involved, most commonly in the distal femur, proximal tibia, and proximal humerus 2, 4-9. Although its incidence is lower than other malignancies, osteosarcoma is characterized by a high mortality rate and early distant metastasis, especially to the lungs. Management of OS consists of surgery along with chemotherapy. The current chemotherapy regimen of the Children's Oncology Group (COG) consists of cisplatin, doxorubicin, methotrexate, and ifosfamide. Despite advances in systemic therapy and supportive care, the overall survival rate for OS, especially in patients with metastatic disease, is low compared with other pediatric neoplasms. Patients diagnosed with localized disease have a 5-year survival rate of 65-70%, compared to those with metastasis, whose 5-year survival rate is dramatically low (20-30%) 10-12.

Insulin-like growth factor binding protein-3 (IGFBP-3), which is the product of a tumor suppressor target gene, can regulate cell proliferation and apoptosis by insulin growth factor (IGF-I)-dependent and IGF-I-independent mechanisms 9, 13. IGFBP-3 controls the bioavailability of IGFs in the extracellular environment, and several studies have established that the IGF-1 signal transduction pathway is involved across species, including nematodes, fruit flies, and mammals interacting with other channels 7-9. Moreover, subcellular localization of local IGFBP-3 plays a role in OS development, because of patients with a low level of IGFBP-3 in OS experience a trend for a shorter survival time than do patients with a high level of IGFBP-3 4.

Graphene is a carbon allotrope consisting of a two-dimensional crystalline lattice of carbon atoms, which are arranged hexagonally. Since the middle of the 20^th^-century graphene-like structures have been studied, but it was not until 2004 that single-layered graphene sheets were successfully isolated. Graphene oxide (GO) is a carbon nanomaterial with potential in material and medical science due to its unique physical, chemical, and mechanical properties. It is a highly oxidized form of chemically modified graphene and is obtained through the oxidation of graphite under strongly acidic conditions. GO has many medical applications, including gene delivery, drug delivery, imaging, cellular probing, cellular differentiation, and photothermal therapy 14.

Previous studies have shown that exposure to GO results in low levels of toxicity in malignant cells such as adenocarcinoma and fibrosarcoma. In the last years, research on the use of GO on OS cells has triggered a number of platforms and stimulated ideas with new aspects that can be tackled in this decade 15-22. Substantially, there is a tremendous need to establish new therapeutic strategies for the treatment of OS for the third decade of the 21^st^ century. GO's relative selectivity in affecting cancer cells but not healthy human cells can potentially provide a new therapeutic strategy for the treatment of cancer 23, 24.

Nuclear factor E2-related factor 2 (Nrf2 or NFE2L2) is a transcription factor that plays a crucial role in the regulation of antioxidant and detoxification enzymes. Nrf2 has protective functions in hepatic inflammation, fibrosis, hepatocarcinogenesis and regeneration, and its expression has been associated with chemoresistance 25.

In this study, we hypothesized that GO has a toxic effect on human OS cancer cells while not affecting normal osteoblasts. The aim of this study was to evaluate the toxicity and underlying mechanism of GO on OS with or without intact *IGF1*/*IGFBP3* genes and normal osteoblasts *in vitro*.

## Materials and Methods

### Materials

GO 1% dispersion was purchased from GO Graphene William Blythe Limited (Essex, England, United Kingdom). Actin, IGF-1, IGFBP3, and Nrf-2 antibodies were purchased from Abcam (Cambridge, MA, USA).

### Cell Cultures

Human OS cell lines, U2OS and SAOS2, and normal osteoblast cell line hFOB1.19 were used for this study. U2OS and SAOS2 were obtained from the cell culture bank of the Japan Health Sciences Foundation (Tokyo, Japan). HFOB1.19 was purchased from the American Type Culture Collection (ATCC, Rockville, MD). The U2OS and SAOS2 cell lines were cultured in DMEM medium (Dulbecco's Modified Eagle Medium). HFOB1.19 was cultured in Ham's F12 DMEM media without phenol red. Fetal bovine serum (FBS) was added to each growth media at a concentration of 10%. The cells were grown in a humidified incubator with 5% carbon dioxide at 37°C.

### CRISPR-Cas9

**Figure 1:**
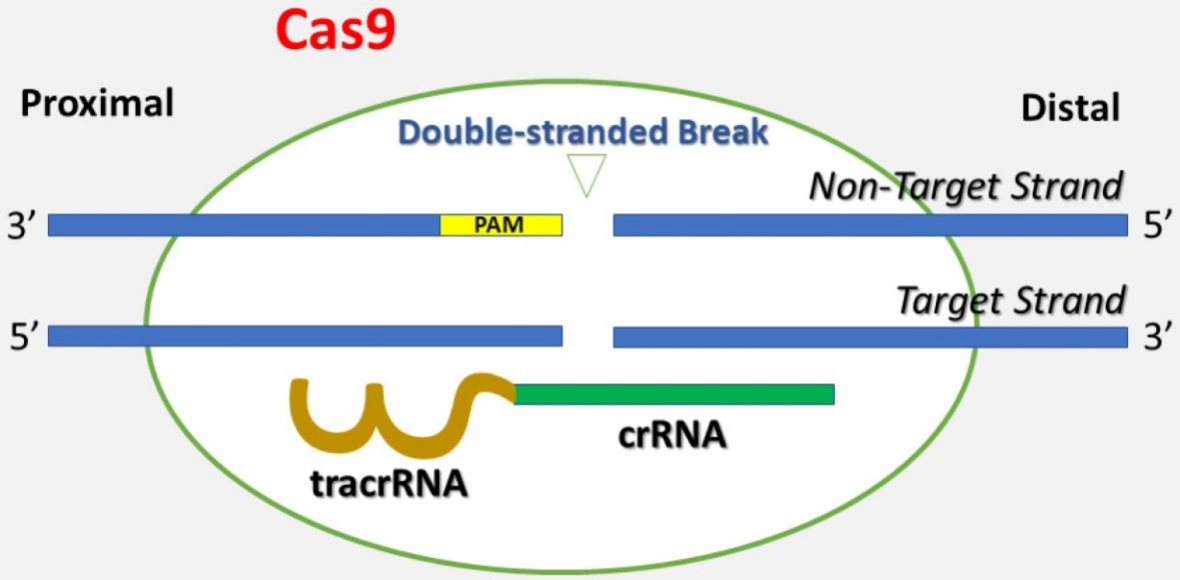
This figure depicts the basis of the CRISPR-Cas9 technique. A single guide RNA (gRNA) consists of CRISPR-derived RNA (crRNA) (green) coupled with a trans-activating CRISPR RNA (tracrRNA) (brown). It targets Cas9 endonuclease to a DNA sequence of interest. Cas9 creates a double-stranded break in the DNA skeleton, prompted by the Protospacer-Adjacent Motif (PAM) (yellow) recognition DNA sequence. Both the target strand and the non-target strands are shown in the figure.

Genetically engineered U2OS cells were established using the CRISPR-Cas9 technique, which is short for clustered regularly interspaced short palindromic repeats and CRISPR-associated protein 9 (**Figure 1**) 26. Briefly, CRISPR-Cas9 is a technique adapted from a naturally occurring genome editing system detected in bacteria. The bacteria catch snippets of DNA from invading viruses and use them to generate DNA segments known as CRISPR arrays. The CRISPR arrays allow the bacteria to "recollect" the viruses. If a virus attacks again, the bacteria produce RNA segments from the CRISPR arrays to specifically target the virus' DNA. Then, the bacteria utilize Cas9 (or a similar enzyme) to cut the DNA apart, which inactivates the virus. Similar to the natural system, the CRISPR-Cas9 method is applied to the lab bench, and a small piece of RNA is created with a short "guide" sequence that binds to a specific target sequence of DNA in a genome. The RNA also links to the Cas9 enzyme. The modified RNA recognizes the DNA sequence of interest, and the Cas9 enzyme cuts the DNA at the targeted location. Once the DNA is reduced, the cell's DNA repair machinery is used to add or delete pieces of genetic material, or to make changes to the DNA by replacing an existing segment with a customized DNA sequence. These cells were modified by one of the authors (AW) using the CRISPR-Cas9 technique Synthego (Menlo Park, California) in the setting of research cooperation. The *IGF1* gene was knocked out in one group of U2OS cells, and the *IGFBP3* gene was knocked out in the other group of U2OS cells. The knockdown of *IGF1* and *IGFBP3* genes was validated via genome sequencing against wild type cells and confirmed by flow cytometry. The engineered cell lines were cultured in DMEM medium (Dulbecco's Modified Eagle Medium) with 10% Fetal bovine serum (FBS). The cells were grown in a humidified incubator with 5% carbon dioxide at 37°C.

### Treatment of Cells with Graphene Oxide

Cell lines were seeded into six-well plates (2×10^5^ cells/well) and cultured for 24 hours, after which the growth medium was removed. The GO stock solution of 1% aqueous dispersion was dissolved in distilled water using one part GO to nine portions of distilled water. The chemical solution was sonicated for 30 minutes and then diluted in the appropriate growth media to concentrations of 25 μg/ml and 50 μg/ml. The cells were then incubated in the respective media containing GO for periods of 30 minutes, 2 hours, 4 hours, 24 hours, and 48 hours.

### Western Blotting

The cultured cells were scraped in PBS and centrifuged. Cell pellets were lysed using 1x RIPA (radio-immuno-precipitation assay) buffer (25% mM Tris HCL (pH 7.6), 150 mM NaCl, 1% Nonidet P-40, 1% sodium deoxycholate and 0.1% SDS) which was supplemented with 1× protease inhibitor. The total protein was quantified using BCA (bicinchoninic acid) protein assay using Pierce ™, Thermo Scientific Ontario, Canada. Western blot analysis was performed using standard techniques. Fifty μg of protein were separated on a 12% SDS PAGE gels and transferred by wet transfer method onto polyvinylidene fluoride (PVDF) membranes. The PVDF membranes were then incubated in tris-buffered saline with 0.1% Tween (TBST) supplemented with 5% non-fat dry milk for 1 hour at room temperature. TBST is a specific mixture of tris-buffered saline (TBS) and Polysorbate 20 (also known as Tween 20 ®). Membranes were probed overnight with anti-actin and anti-Nrf2 antibodies diluted in TBST at a concentration of 1:1000. The antibodies were then probed the next day with an HRP-conjugated secondary antibody at room temperature for 1 hour. The western blots were visualized using enhanced chemiluminescence (ECL) Western blotting detection reagents using a luminol-based substrate for the detection of horseradish peroxidase (HRP) on immunoblots and developed on Kodak film.

### Morphology

Cell lines were incubated in 0, 25, and 50 μg/ml of GO for 30 minutes, 2 hours, 4 hours, 24 hours, and 48 hours. The cells were washed with PBS, and images were taken using a Zeiss Axioskop microscope and camera, along with Zeiss Axiovision software.

### Apoptosis Detection

Apoptosis detection was conducted using the eBioscience Annexin V-FITC Apoptosis Detection Kit purchased from ThermoFisher. Cells were harvested at 30 minutes, 2 hours, 4 hours, 24 hours, and 48 hours and washed with PBS after treatment with GO at concentrations of 0, 25, and 50 μg/ml. The cells were collected using ethylenediaminetetraacetic acid (EDTA) free trypsin and then resuspended in PBS. After which the cells were centrifuged. The cells were then stained with 5 μl fluorescein isothiocyanate (FITC)-Annexin V, incubated at room temperature protected from light and then stained with 10 μl propidium iodide (20 μg /ml). Apoptosis was tested and analyzed using the flow cytometry assay for the Muse Cell Analyzer according to the manufacturer's instructions.

### Reactive Oxygen Species Detection

Reactive oxygen species (ROS) detection was conducted using the Invitrogen Total ROS assay kit purchased from Thermo Fisher Scientific (Waltham, MA, USA). A 500X stock solution of ROS assay stain was made by adding 40 μl of DMSO into the vial of ROS assay stain concentrate. The ROS assay stain was added to the cells in culture media at a rate of 2 μl for every 1 ml of media resulting in a final concentration of 1X. The cells were incubated for 2 hours using a 37°C incubator with 5% CO, after which these cells were treated with GO. The cells were collected using ethylenediaminetetraacetic acid (EDTA) free trypsin and then resuspended in PBS. Afterwards the cells were centrifuged. ROS was tested and analyzed using the Muse Cell Analyzer according to the manufacturer's instructions.

### Statistical Analysis

Experiments for apoptosis detection and ROS detection using flow cytometry were conducted in triplicates for each concentration of GO and incubation time for each cell line. The mean values were then calculated. Analysis of Variance (ANOVA) was calculated using the online ANOVA calculator developed by Dr. Lawrence Turner of Southwestern Adventist University and validated by GraphPad Prism 7.05 (GraphPad Software, Inc., CA, US).

## Results

### Graphene oxide induces morphological changes in the osteosarcoma cell lines

**Figure 2:**
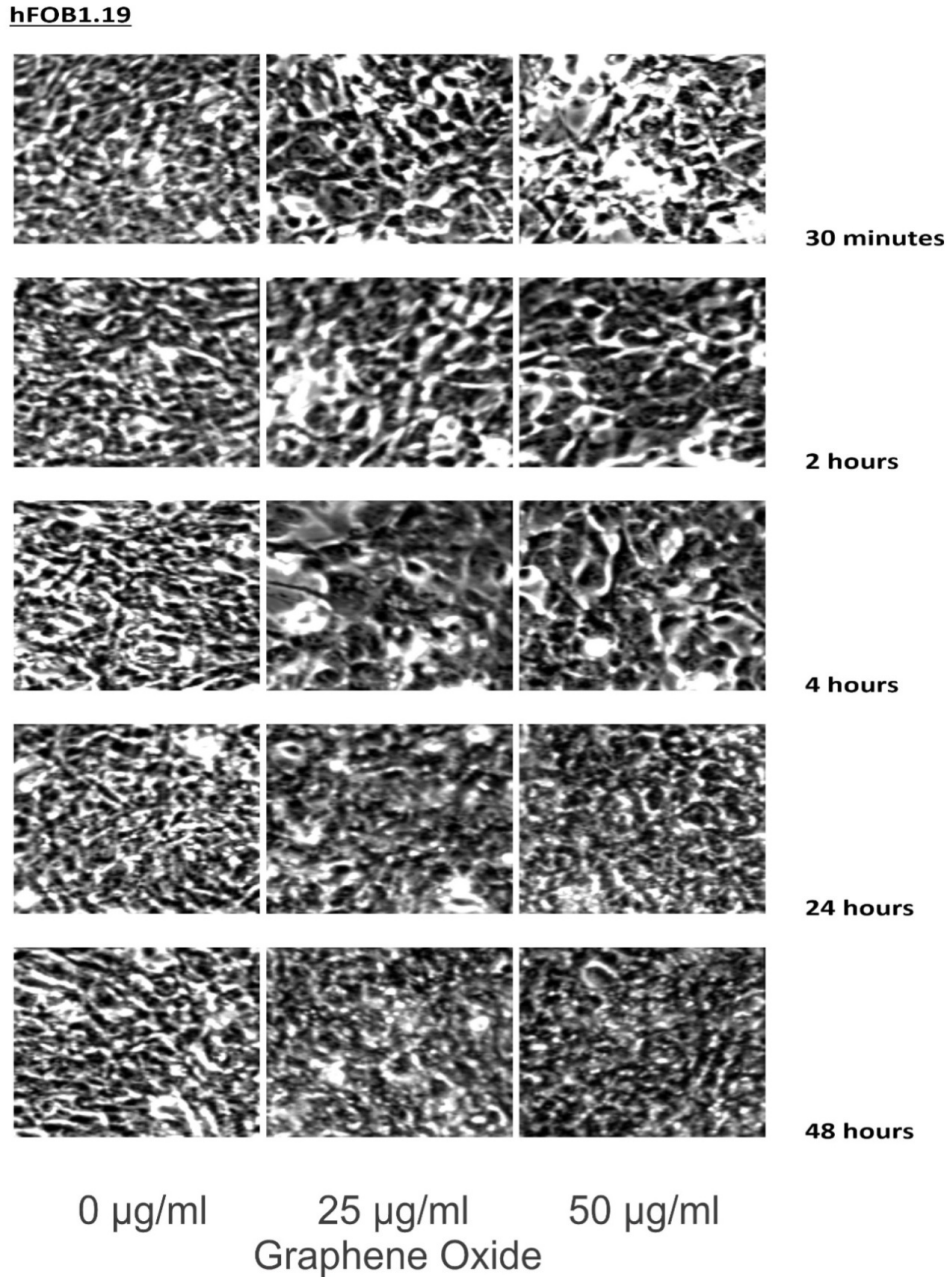
These figures illustrate the morphological changes of hFOB1.19 on exposure to 25 µg/ml and 50 µg/ml, for exposure times from 30 minutes to 48 hours. The morphological changes were more visible with increasing concentration and time of incubation with GO. These morphological effects were observed in the OS cell lines U2OS and SAOS2. However, only minimal morphological changes were observed in the hFOB1.19 cells that were treated with GO under the same conditions as the specified cancer cell lines (see text for details).

**Figure 3:**
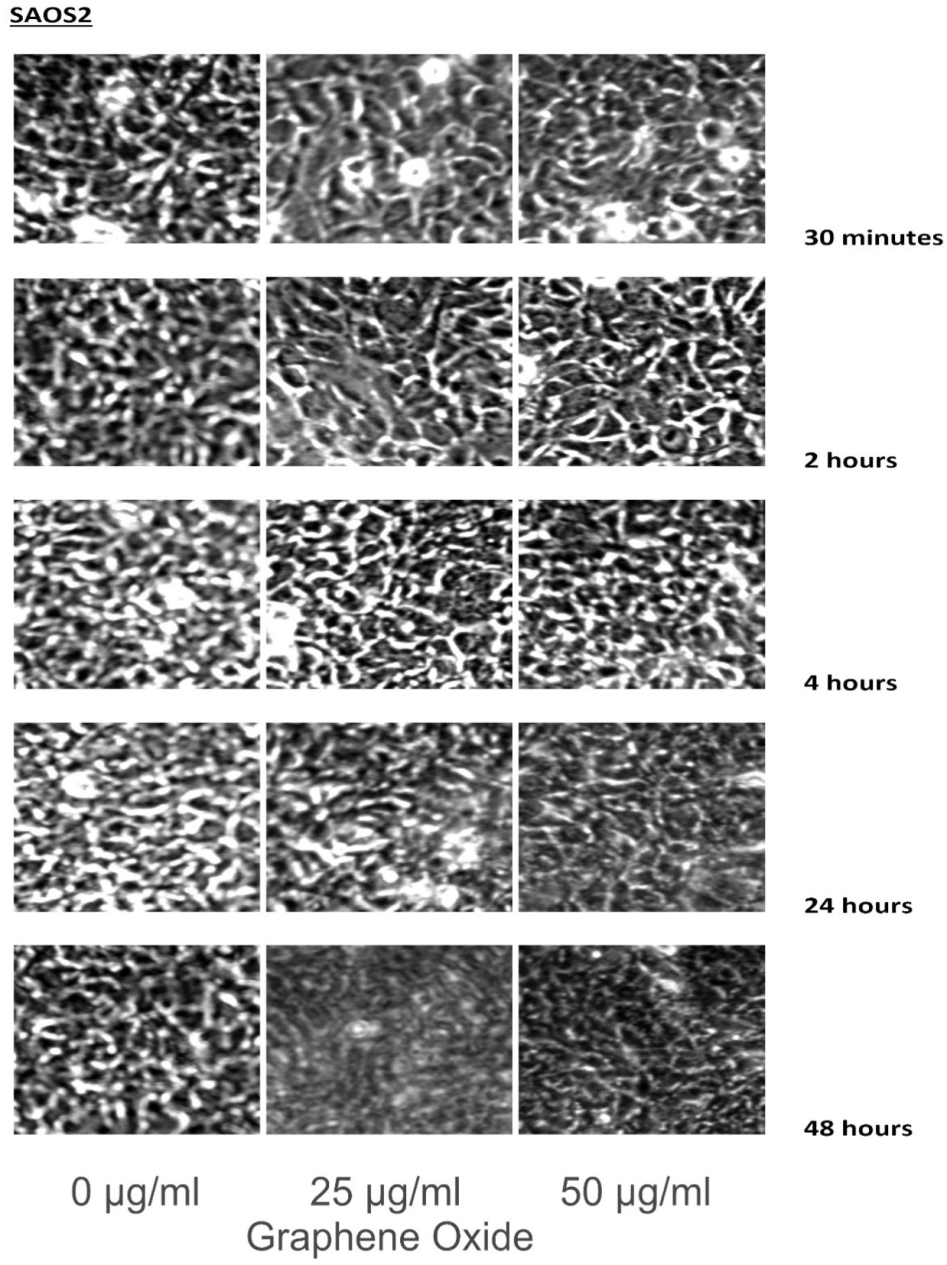
These figures illustrate the morphological changes of SAOS2 on exposure to 25 µg/ml and 50 µg/ml, for exposure times from 30 minutes to 48 hours. The morphological changes were more visible with increasing concentration and time of incubation with GO. These morphological effects were observed in the OS cell lines U2OS and SAOS2. However, only minimal morphological changes were observed in the hFOB1.19 cells that were treated with GO under the same conditions as the specified cancer cell lines (see text for details).

**Figure 4:**
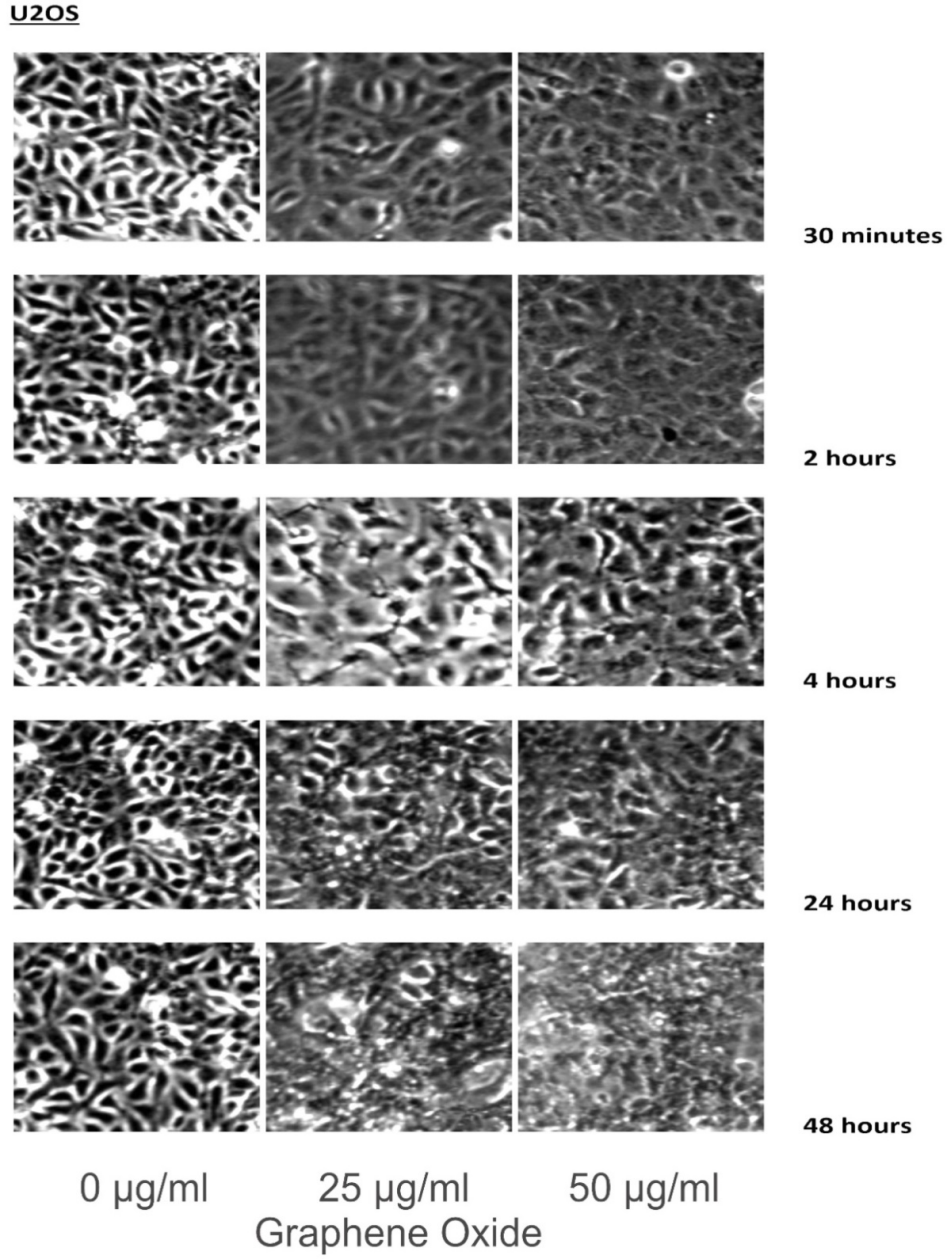
These figures illustrate the morphological changes of U2OS on exposure to 25 µg/ml and 50 µg/ml, for exposure times from 30 minutes to 48 hours. The morphological changes were more visible with increasing concentration and time of incubation with GO. These morphological effects were observed in the OS cell lines U2OS and SAOS2. However, only minimal morphological changes were observed in the hFOB1.19 cells that were treated with GO under the same conditions as the specified cancer cell lines (see text for details).

The detection of the effect of GO on the various cell lines was examined through an inverted microscope. It was observed that many cells exhibited changes in their orientation, while some cells became disordered and loosely adherent to their substrates. There were numerous floating cells and cellular debris, which indicated the presence of dead cells. GO nanomaterials were visible and adherent to the cell surface membrane of the cells, which supported GO's interaction with cell membranes. This observation was more pronounced in the cells that were incubated with GO for 24 hours and 48 hours. Individual cells became swollen and developed large cytoplasmic vacuoles. The morphological changes were more visible with increasing concentration and time of incubation with GO. These morphological effects were observed in the OS cell lines U2OS and SAOS2. However, only minimal morphological changes were observed in the hFOB1.19 cells that were treated with GO under the same conditions as the specified cancer cell lines (**Figures 2-4**). **Figures 2** to **4** illustrate the morphological changes of the various cell lines on exposure to 25 μg/ml and 50 μg/ml, for exposure times from 30 minutes to 48 hours.

### Graphene Oxide Induces Apoptosis in osteosarcoma cells but not in normal osteoblasts *in vitro*

**Figure 5:**
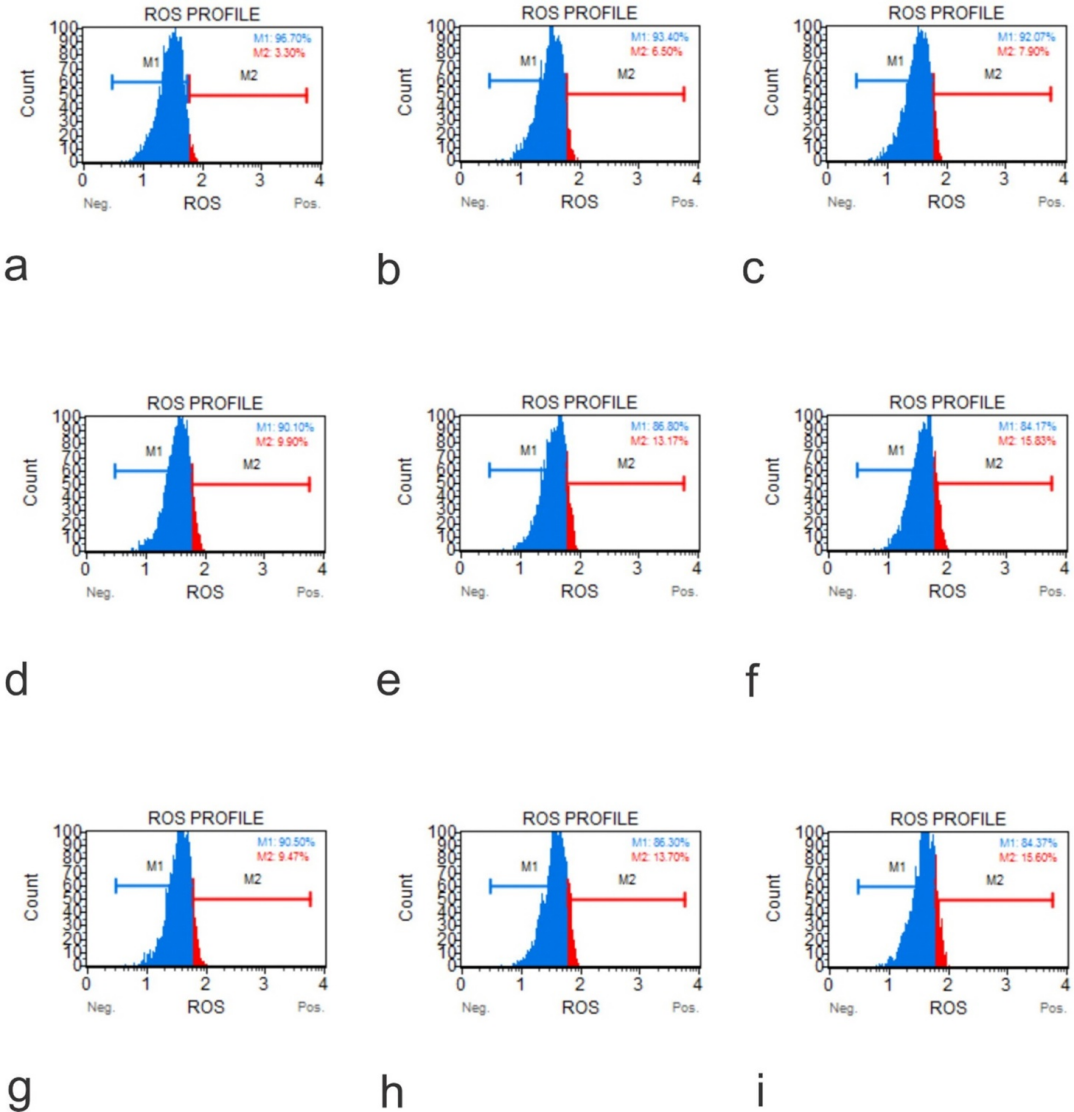
Annexin-Apoptosis Profile on Native Cells. The apoptotic activity is different according to the native cell lines (**a**, Flow Cytometry Image for Apoptosis for HFOB 1.19 48 hours 0 µg /ml; **b**, Flow Cytometry Image for Apoptosis for HFOB 1.19 24 hours 50 µg /ml; **c**, Flow Cytometry Image for Apoptosis for HFOB 1.19 48 hours 50 µg /ml; **d**, Flow Cytometry Image for Apoptosis for SAOS2 48 hours 0 µg /ml; **e**, Flow Cytometry Image for Apoptosis for SAOS2 24 hours 50 µg /ml; **f**, Flow Cytometry Image for Apoptosis for SAOS2 48 hours 50 μg /ml; **g**, Flow Cytometry Image for Apoptosis for U20S 48 hours 0 μg /ml; **h**, Flow Cytometry Image for Apoptosis for U20S 24 hours 50 μg /ml; **i**, Flow Cytometry Image for Apoptosis for U20S 48 hours 50 µg /ml).

The cytotoxic effects of GO on cell lines hFOB1.19, SAOS-2 and U2OS, were tested at two doses over a 48-hour time course using an Annexin V-FITC Apoptosis Kit (**Figure 5**). The results showed that the application of GO increased the percentage of cells that underwent total apoptosis (early and late apoptosis) in a dose-dependent manner for the OS cell lines (SASOS2 and U2OS). Increased apoptosis was also observed as the treatment time of the cancer cell lines with GO was increased. There was no significant apoptosis detection in hFOB1.19 (normal osteoblast) cell line concerning the concentration or time of treatment. There was no significant difference in the percentage of live cells or the percentage of apoptotic cells for the normal osteoblast cell line hFOB1.19. The rate of living cells was approximately 96% (apoptotic cells between 2.95% to 4.61%) for the untreated controls, as well as for hFOB1.19 cells treated with GO and incubated for periods from 30 minutes to 24 hours. The percentage of living cells decreased to 94.5% and 94.3%, while the rate of apoptotic cells increased to 5.1% and 5.3% at 48 hours for GO concentrations of 25 μg/ml and 50 μg/ml, respectively. GO had a significant effect on the rate of total apoptosis in the OS cell lines (SAOS2 and U2OS). The observed trend in apoptosis was an increase as the time of exposure to GO was increased. This increase was dose dependent. This trend was noted for all times of exposure and concentrations of GO. For example, at 48 hours exposure to 50 μg/ml of GO, the total percentage of apoptotic cells for the OS cell lines of SAOS and U2OS were 24.08% and 26.28%, respectively.

### Graphene Oxide induced reactive oxygen species in osteoblasts and osteosarcoma cells

**Figure 6:**
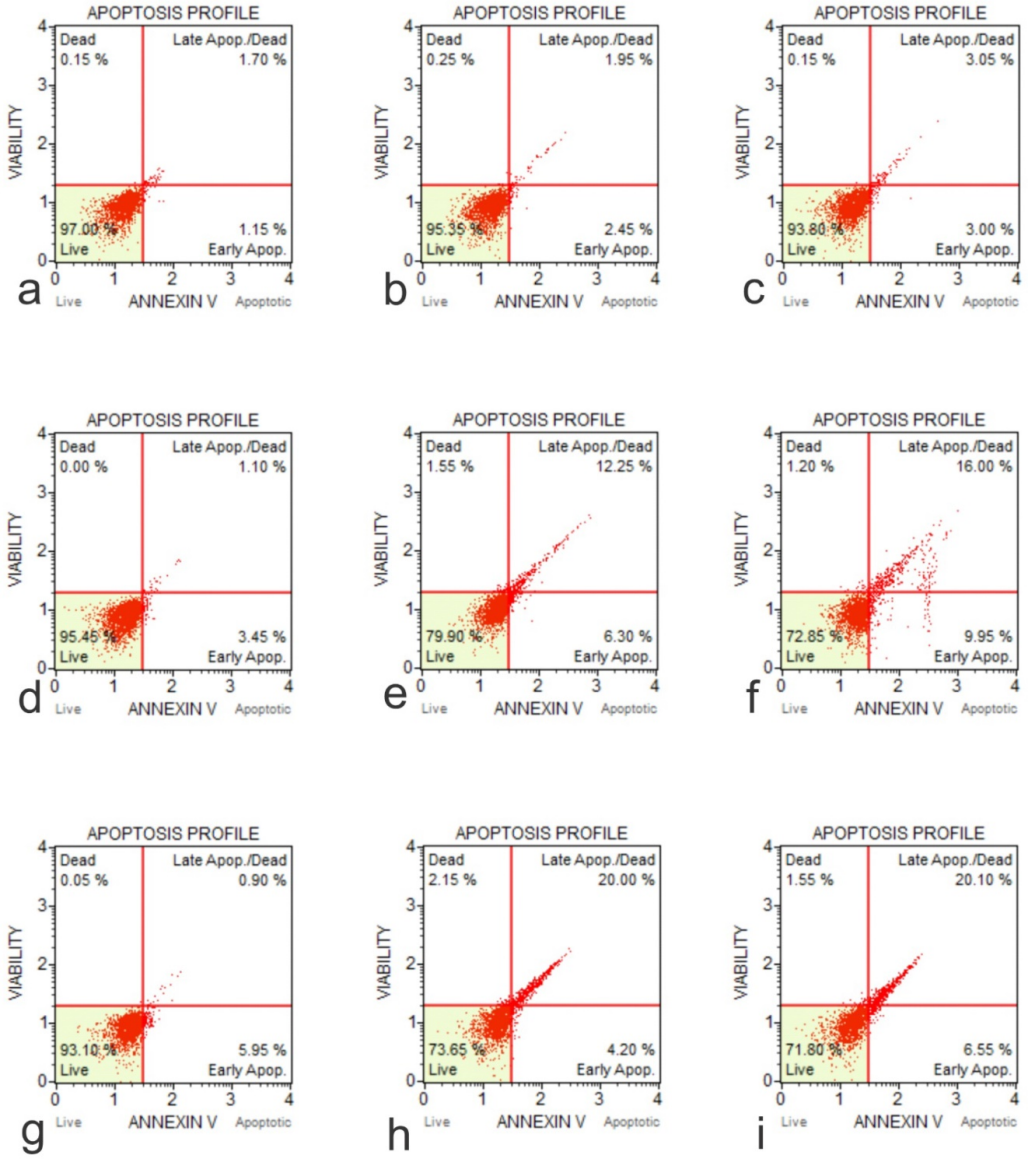
ROS Profile on Native Cells (**a**, Muse Analyzer Image for Reactive Oxygen Species for HFOB 1.19 48 hours 0 µg /ml; **b**, Muse Analyzer Image for Reactive Oxygen Species for HFOB 1.19 24 hours 50 µg /ml; **c**, Muse Analyzer Image for Reactive Oxygen Species for HFOB 1.19 48 hours 50 µg /ml; **d**, Muse Analyzer Image for Reactive Oxygen Species for SAOS2 48 hours 0 µg /ml; **e**, Muse Analyzer Image for Reactive Oxygen Species for SAOS2 24 hours 50 µg /ml; **f**, Muse Analyzer Image for Reactive Oxygen Species for SAOS2 48 hours 50 µg /ml; **g**, Muse Analyzer Image for Reactive Oxygen Species for U2OS 48 hours 0 µg /ml; **h**, Muse Analyzer Image for Reactive Oxygen Species for U2OS 24 hours 50 µg /ml; **i**, Muse Analyzer Image for Reactive Oxygen Species for U2OS 48 hours 50 µg /ml).

The production of ROS induced by GO on cell lines hFOB1.19, SAOS-2, and U2OS were tested at two doses over a 48-hour time course using an Invitrogen Total ROS Kit. The results showed that the application of GO increased the percentage of cells which tested positive for ROS in a dose and concentration-dependent manner. This aspect was seen for all the cells tested. The rate of ROS positive cells was higher in the osteosarcoma cells than the normal osteoblasts for corresponding exposure time and concentrations (**Figure 6**).

### Nrf-2 was highly expressed in osteoblastoma, but low expressed in osteosarcoma cell lines

**Figure 7:**
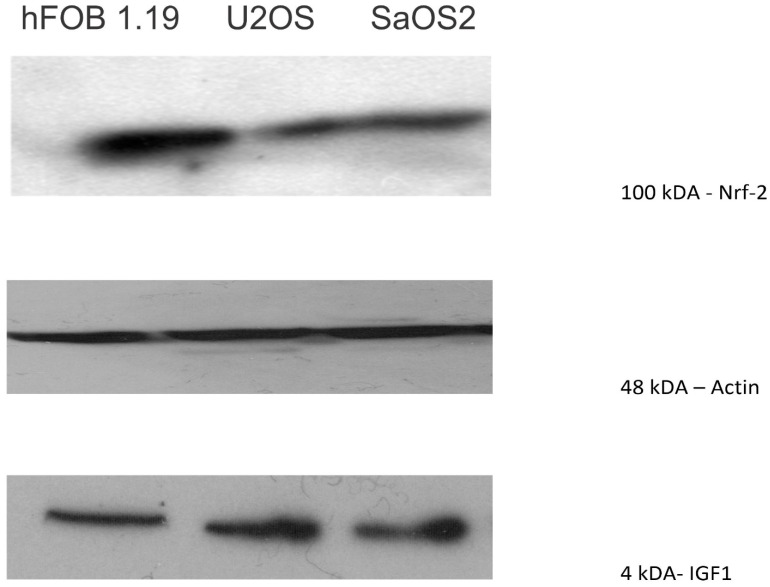
Western blot showing that Nrf2, a transcription factor that protects against oxidative damage, was moderately expressed in U2OS and SAOS2 (100 kDA). The normal osteoblast cell line, hFOB1.19, showed a higher level of expression of Nrf2 than the OS cell lines.

Nrf2, a transcription factor that protects against oxidative damage, was moderately expressed in U2OS and SAOS2, with expression mainly observed at 100 kDA. The normal osteoblast cell line, hFOB1.19, showed a higher level of expression of Nrf2 than the OS cell lines (**Figure 7**).

### Knockout of *IGF1* and *IGFBP3* genes increased the rate of apoptosis in U2OS cells

**Figure 8:**
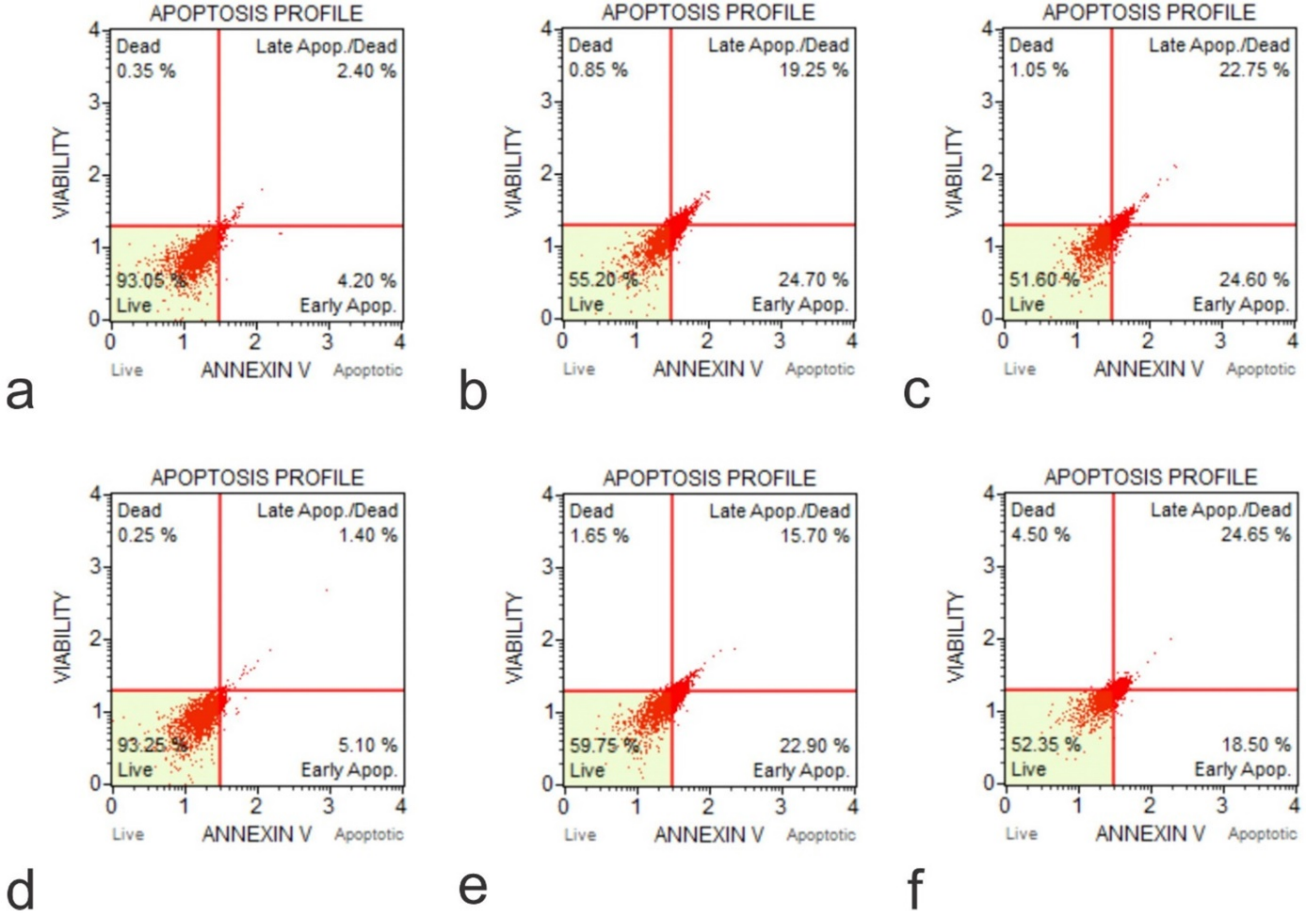
Annexin-Apoptosis Profile on KO Cells (**a**, Flow Cytometry Image for Apoptosis for U20S IGF1 KO 48 hours 0 µg /ml; **b**, Flow Cytometry Image for Apoptosis for U20S IGF1 KO 24 hours 50 µg /ml; **c**, Flow Cytometry Image for Apoptosis for U20S IGF1 KO 48 hours 50 µg /ml; **d**, Flow Cytometry Image for Apoptosis for U20S IGFBP3 KO 48 hours 0 µg /ml; **e**, Flow Cytometry Image for Apoptosis for U20S IGFBP3 KO 24 hours 50 µg /ml; **f**, Flow Cytometry Image for Apoptosis for U20S IGFBP3 KO 48 hours 50 µg /ml).

**Figure 9:**
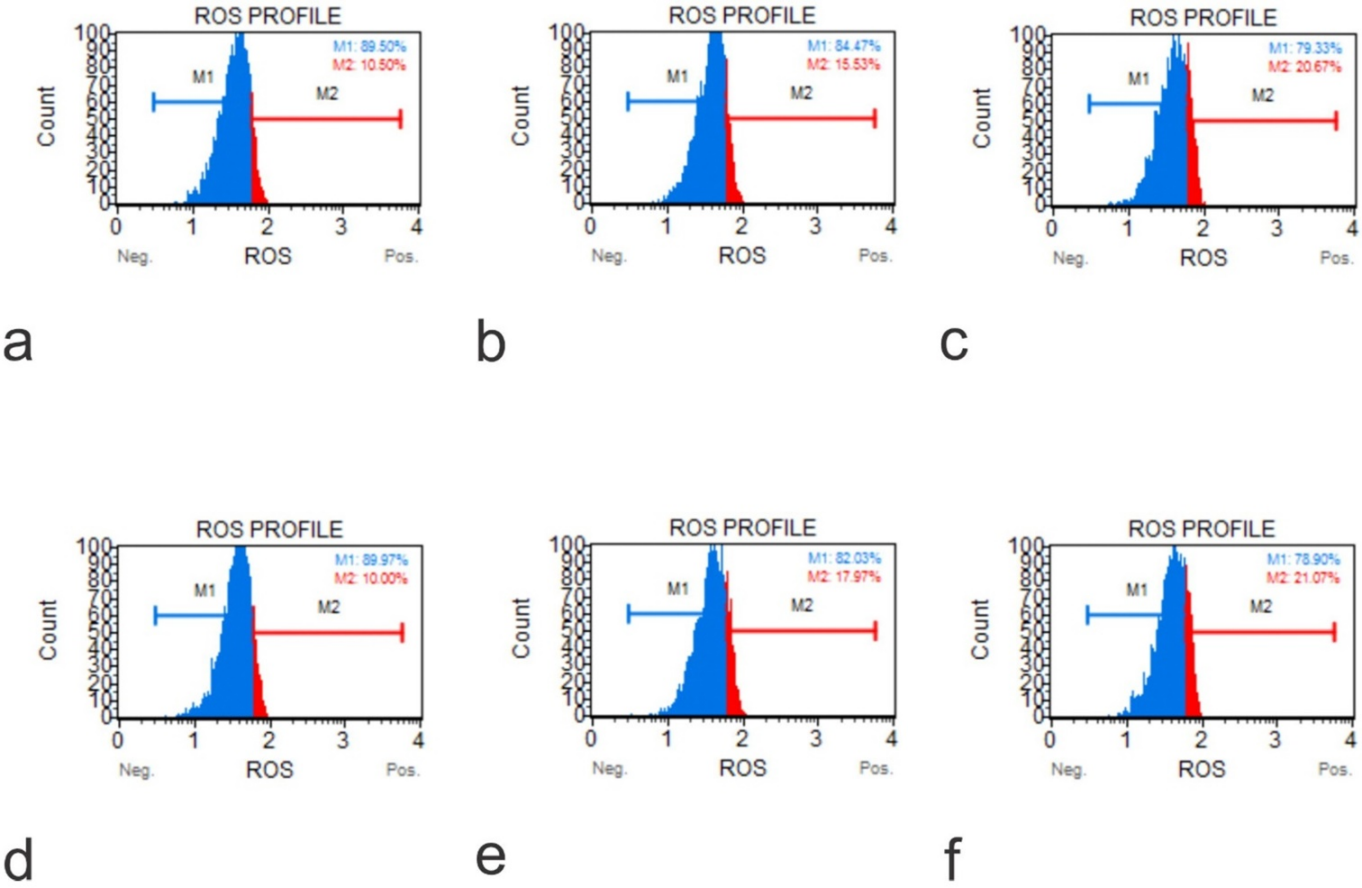
ROS Profile on KO Cells (**a**, Muse Analyzer Image for Reactive Oxygen Species for U2OS IGF1 48 hours 0 µg /ml; **b**, Muse Analyzer Image for Reactive Oxygen Species for U2OS IGF1 24 hours 50 µg /ml; **c**, Muse Analyzer Image for Reactive Oxygen Species for U2OS IGF1 48 hours 50 µg /ml; **d**, Muse Analyzer Image for Reactive Oxygen Species for U2OS IGF1 48 hours 0 µg /ml; **e**, Muse Analyzer Image for Reactive Oxygen Species for U2OS IGF1 24 hours 50 µg /ml; **f**, Muse Analyzer Image for Reactive Oxygen Species for U2OS IGF1 48 hours 50 µg /ml).

**Figure 10:**
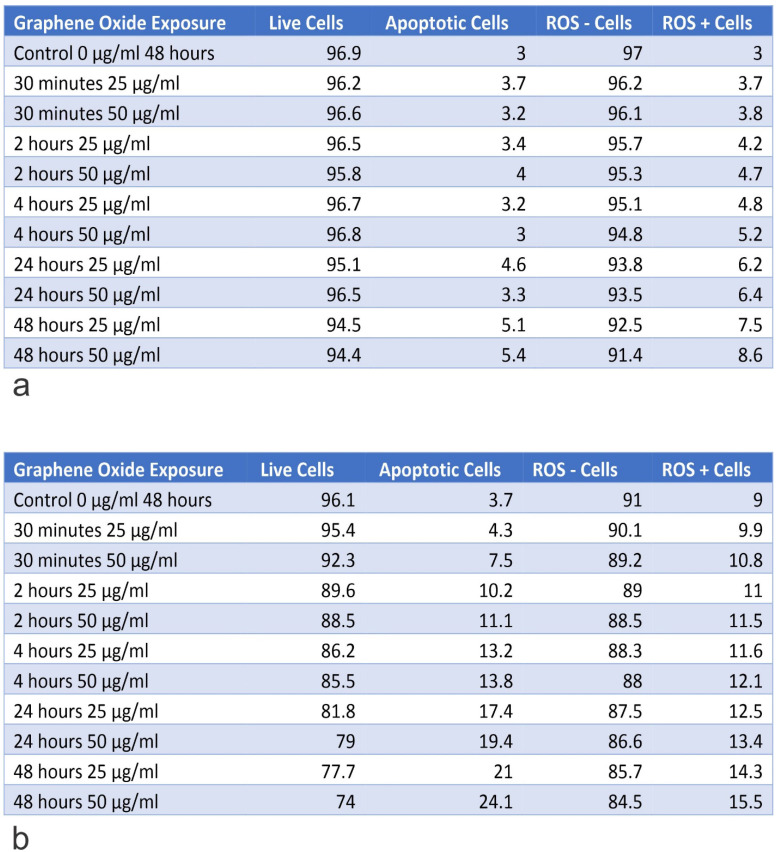
Table A shows the percentage of live and apoptotic cells, as well as, the percentage of ROS+ and ROS- cells for hFOB1.119 on exposure to 25 µg/ml and 50 µg/ml, for exposure times from 30 minutes to 48 hours. Table B shows the percentage of live and apoptotic cells, as well as, the percentage of ROS+ and ROS- cells for SAOS2 on exposure to 25 µg/ml and 50 µg/ml, for exposure times from 30 minutes to 48 hours.

**Figure 11:**
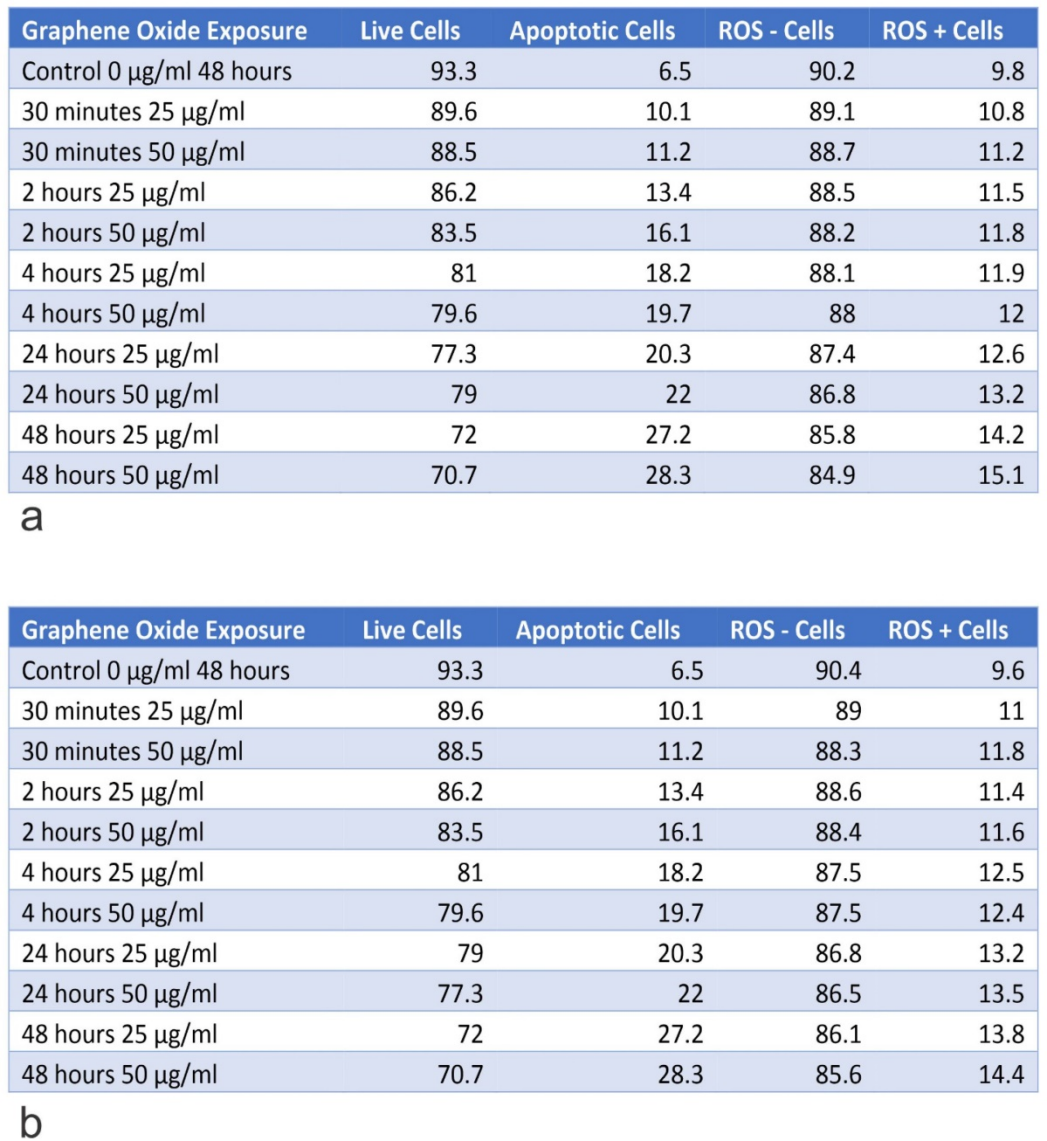
Table A shows the percentage of live and apoptotic cells, as well as, the percentage of ROS+ and ROS- cells for U2OS on exposure to 25 µg/ml and 50 µg/ml, for exposure times from 30 minutes to 48 hours. Table B shows the percentage of live and apoptotic cells, as well as, the percentage of ROS+ and ROS- cells for U2OS (Engineering Control) on exposure to 25 µg/ml and 50 µg/ml, for exposure times from 30 minutes to 48 hours.

**Figure 12:**
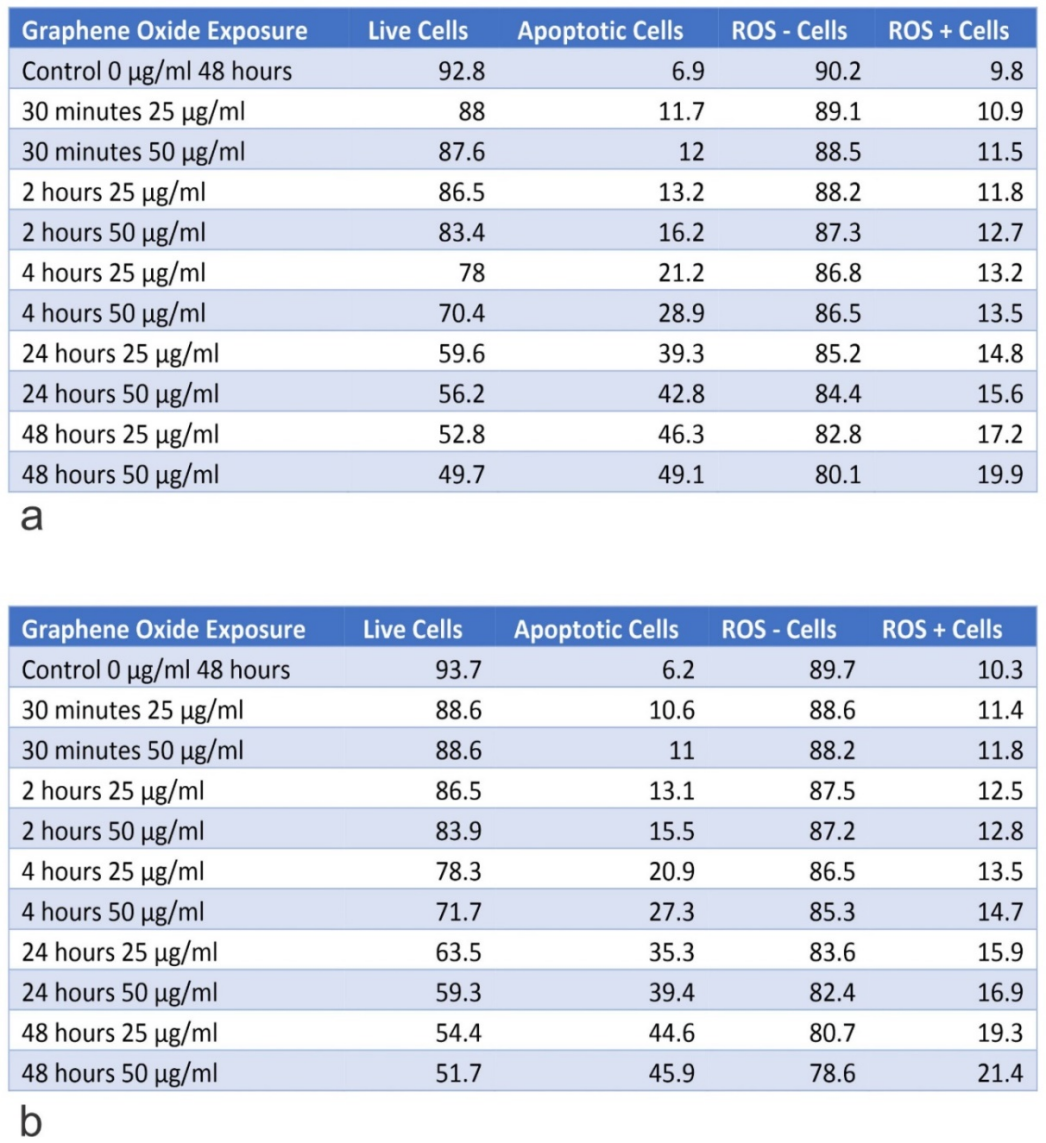
Table A shows the percentage of live and apoptotic cells, as well as, the percentage of ROS+ and ROS- cells for the knockout of U2OS IGF-1 on exposure to 25 µg/ml and 50 µg/ml, for exposure times from 30 minutes to 48 hours. Table B shows the percentage of live and apoptotic cells, as well as, the percentage of ROS+ and ROS- cells for knockout of U2OS IGF-BP3 on exposure to 25 µg/ml and 50 µg/ml, for exposure times from 30 minutes to 48 hours.

The *IGF1* knockout U2OS and *IGFBP3* knockout U2OS cells were treated with GO at concentrations of 25 μg/ml and 50 μg/ml for periods of 30 minutes, 2 hours, 4 hours, 24 hours, and 48 hours. Subsequently, the cytotoxic effect was tested using an Annexin V-FITC Apoptosis detection kit (**Figure 8**). Results showed that GO had a higher cytotoxic effect on the *IGF1* knockout U2OS and *IGFBP3* knockout U2OS cells than it did on the unmodified U2OS cells. The application of GO increased the percentage of cells that underwent total apoptosis (early and late apoptosis) in a dose-dependent manner for U2OS, *IGF1* knockout U2OS, and *IGFBP3* knockout U2OS cells. The knockdown of *IGF1* and *IGFBP3* also increased the percentage of cells that were positive for ROS. These differences were more significant with increased exposure time and concentration of GO. GO was slightly more effective in inducing apoptosis in *IGF1* knockout U2OS than *IGFBP3* knockout U2OS for corresponding exposure times and concentrations. The knockout of *IGF-1* and *IGFBP3* increased the cytotoxic effect of GO on OS (**Figure 9**). **Figures 10** to **12** illustrate the toxicity of GO on the various cell lines at different concentrations and incubation times.

## Discussion

Prior research has shown that GO can induce inflammation, oxidative stress, DNA damage, apoptosis, autophagy, and necrosis 1. Also, GO reduces cell viability and increases mitochondrial dysfunction, lactate dehydrogenase expression, and generation of ROS. In our study, we investigated the effect of GO on OS and normal osteoblasts *in vitro*. The concentrations of GO used for this experiment were 25 μg/ml and 50 μg/ml, with cells being incubated from 30 minutes to 48 hours. The morphological changes, ROS generation, and rate of apoptosis were evaluated for cells exposed to GO and compared with cells cultured under the recommended conditions. The percentage of live cells and apoptotic cells were then measured after the required incubation time. The percentage of total apoptotic cells was the combined total of cells in early and late apoptosis. The percentage of cells which were positive and negative for ROS were measured after the required incubation time. Total ROS include peroxides, superoxide, hydroxyl radical, singlet oxygen, and alpha oxygen.

GO is a highly oxidized form of chemically modified graphene, obtained by the oxidation of graphite under concentrated acidic conditions. The structure of GO consists of a basal plane and edges with oxygen-containing functional groups. The oxygen-containing functional groups include hydroxyl, epoxy, and carbonyl groups 27-30. GO has many unique physiochemical properties such as electronic properties, which are associated with its unique gapless conical band structure, high mechanical strength, flexibility, thermal conductivity, and stability, with a wide range of light absorption. These properties allow for GO to potentially have a wide range of applications. However, there is an irreversible agglomeration or restacking of graphene sheets due to the robust van der Waals interactions and the high inter-sheet junction contact resistance found between isolated graphene sheets. This setting results in a diminished accessible surface area, in addition to suppression of the high conductivity and mechanical strength of individual graphene sheets. This challenge of restacking is overcome effectively by constructing three-dimensional networks. These 3D networks have various morphologies, structures, and properties, and include graphene foams, graphene sponges, and graphene aerogels 30-32. GO has been shown to have some cytotoxic effect on cancer cells while having an apparent minimal impact on non-cancerous cells 30-32.

GO was observed to induce morphological changes in the cell membranes of the cancer cell lines. Previous research suggests that GO selectively targets the microtubules of the cytoskeleton. It is the cytoskeleton that helps the cell to maintain its shape and internal organization 33-37. The changes in the morphology of the cell membrane are due to damage to the cytoskeleton caused by GO 38-42. This compound interacts with the cell membrane by adhesion. It inserts into the lipid bilayer to be internalized into the cell. Strong dispersion interactions between graphene and lipid tail carbons are believed to result in depleting lipid density in the cell membrane, which leads to water-permeable pores. Morphologically, SAOS2 and U2OS were affected by GO, while there were minimal changes noted in the morphology of hFOB1.19 cells on exposure to GO at 25 μg/ml and 50 μg/ml over the different time periods when compared to the untreated control. The morphological changes of the various cell lines on exposure to 25 μg/ml and 50 μg/ml, for exposure times from 30 minutes to 48 hours were compared to cells grown under the recommended conditions 20, 32, 43.

**Apoptosis** is a gene-controlled process of programmed cell death that maintains cell homeostasis. It is characterized by morphological changes such as cell shrinkage, nuclear condensation, nuclear fragmentation, and the appearance of apoptotic bodies. The complex process of apoptosis can be activated through several signalling pathways. GO exposure increases intracellular ROS generation, including mitochondria produced superoxide 20, 24, 32, 44. Studies have demonstrated that mitochondria act as a primary control point for regulating apoptosis 45. Treatment with GO causes mitochondrial damage and accelerates apoptosis by generating oxidative stress 20, 32, 43, 46. GO has the therapeutic potential for the treatment of malignancies including OS since it targets cell cycle arrest and causes the induction of apoptosis. GO induces cell cycle arrest at the G0/G1 phase. The ability to induce apoptosis and cause cell cycle arrest are both properties that are exhibited in some anti-cancer treatments 43.

Mitochondria act as the primary source of cellular **ROS**. The excessive generation of ROS results in mitochondria dysfunction. The accumulation of intracellular ROS induces cell cycle arrest at the G1 or G2/M phase, impeding cell proliferation. Research has demonstrated that cancer cells have an abnormal increase in ROS levels. This setting results in the cancer cells being more vulnerable to further oxidative stress 47, 48.

ROS is one of the most chemically important molecules since it acts as a second messenger in cell signaling and plays essential roles in the regulation of cellular functions. A moderate increase in ROS promotes cell differentiation and proliferation, while excessive ROS accumulation causes oxidative stress and cellular damage. Malignant cells have increased ROS levels. Tumors are characterized by rapid growth primarily attributed to enhanced activation of growth factors and intracellular signaling pathways related to proliferation, which may be promoted by ROS 47, 49. The targeting ROS may provide a novel cancer therapeutic strategy. The cancer-killing potential of ROS can be exploited by either inducing the generation of ROS directly in tumor cells or inhibiting the anti-oxidative enzyme system of tumor cells. GO acts by producing ROS directly in tumor cells. Many anti-tumor agents reportedly disturb the electron transport chain (ETC), leading to increased electronic leakage from the chain and elevated ROS production. The ETC components such as complex I and II are direct targets of ROS promoting agents 47, 49, 50.

**IGF** plays a vital role in the growth and development of tissues. Previous studies have demonstrated that an increased level of circulating IGFs is associated with an increased risk of cancers such as prostate, breast, colorectal, and ovarian cancer. IGF-1 is a member of the IGF family and is involved in the regulation of cell survival, proliferation, and cell distribution. There is evidence that IGF-1 related pathways are involved in osteosarcoma progression. There is downstream of signaling pathways such as the MAPK, PI3K-Akt, and NF-kB pathways 51, 52.

IGFBP3 is expressed in most body tissues and acts as an antagonist of IGF binding to the signal-transducing type 1 receptor (IGF1R). This setting blocks the proliferative and cell survival effects, which are elicited by its activation. IGFBP-3 is a critical regulatory molecule in the IGF system and can function in a tissue-specific manner as either a tumor suppressor or tumor promoter. The IGF binding proteins are key regulatory molecules in the IGF system, as aberrations in their expression or function are associated with the development of a wide range of cancers 53. IGFBP3 controls the bioavailability and bioactivity of IGF-1 exquisitely. The IGF system has been linked in the development and progression of a wide range of tumors, as it plays an important role in tumorigenesis by virtue of its proliferative and anti-apoptotic effects. IGF-1 is an attractive therapeutic target since IGF-1 is involved in the proliferation, survival, invasion, and metastasis of tumors. Knocking out *IGF1* and *IGFBP3* increased the cytotoxic effect of GO on U2OS cell line. For example, at 48 hours of exposure to 50 μg/ml of GO, the total percentage of apoptotic cells for the U2OS *IGF-1* knocked out, and U2OS *IGFBP3* knocked out were 49.1% and 45.9%, respectively compared to 26.28% for U2OS. Knocking out *IGF-1* was slightly more effective than knocking out the *IGFBP3* gene 51, 52. The results from flow cytometry and morphological changes support the hypothesis that GO has a toxic effect on human cancer cells but not on healthy cells. This aspect was evident in the increased rate of apoptosis, ROS, and morphological changes observed in the osteosarcoma cell lines (SAOS2 and U2OS) compared to the standard osteoblast cell line (hFOB1.19).

Currently, there are several synthetic methods used to fabricate 3D graphene networks. These strategies rely on either direct growth from a carbon source or assembly of GO/graphene sheets. The 3D structure of the network provides a high specific surface area, powerful mechanical strength along with fast mass and electron kinetic transport. The properties of GO allow its utilization in many medical applications, including gene delivery, drug delivery, imaging, cellular probing, cellular differentiation, and photothermal therapy.

In conclusion, the study demonstrated the cytotoxic effects of GO on OS and specifically on *IGF-I* or *IGFBP3* knocked out OS cell lines. Our data support a further preclinical development towards its potential to use as a treatment option for OS. Our results may support the possibility of GO as an adjuvant therapeutic option for OS. There is a need for further research, including *in vivo* studies, to investigate cytotoxic effects of GO on OS and non-bony cells.

## Abbreviations

GO: graphene oxide
IGF: insulin growth factor
IGFBP3: insulin growth factor binding protein 3
OS: osteosarcoma
